# Association of the electrical parameters and photosynthetic characteristics of the tea tree manifests its response to simulated karst drought

**DOI:** 10.1080/15592324.2024.2359258

**Published:** 2024-06-03

**Authors:** Peng Wei, Haitao Li, Yanyou Wu, Cheng Zhang

**Affiliations:** a College of Forestry, Guizhou University, Guiyang, Guizhou, China; b State Key Laboratory of Environmental Geochemistry, Institute of Geochemistry, Chinese Academy of Sciences, Guiyang, China; c Engineering Technology Research Center for Protection and Detection of Germplasm Resources of Karst-Adaptable Crops, Guizhou Vocational College of Agriculture, Guiyang, Guizhou, China; d School of Public Health, Guizhou Medical University, Guiyang, Guizhou, China

**Keywords:** Karst, drought and rewatering, photosynthesis, intracellular water, *Camellia sinensis* (L.)

## Abstract

Tea plantations in Karst regions suffer from the serious effects of frequent temporary karst droughts, leading to a decline in tea production and quality in the region. The close relationship between growth and electrical parameters of plants, including physiological capacitance, resistance and impedance, can be used to accurately monitor their plant water status online, quickly, accurately, timely and nondestructively. In this study, three tea tree cultivars of Zhonghuang No.2 (ZH), Wuniuzao (WNZ), and Longjing 43 (LJ) with different levels of drought resistance were selected as experimental materials, and experiments were carried out under controlled conditions according to control (soil water content of 40–45%, D0), (keeping D0 no watering to 5 days, D5), (keeping D0 no watering to 10 days, D10), (the first day after D10 is rehydrated to D0 is regarded as R1) and (the fifth day after D10 rehydration to D0 is regarded as R5), to determine intracellular water metabolism and nutrient translocation characteristics based on intrinsic electrical parameters. The photosynthetic characteristics and chlorophyll fluorescence parameters were also determined to investigate the response of water metabolism to simulated karst drought in the three tea tree cultivars. The results indicated that the water metabolism patterns responded to environmental water changes with a medium water-holding capacity, medium water transport rate, and low water-use efficiency, and the nutrient patterns in those tea tree varieties demonstrated with a high nutrient flux per unit area, low nutrient transfer rate, and high nutrient transport capacity. After rehydration, only the electrical characteristics of WNZ returned to the D0 levels, but the net photosynthetic rate of all varieties returned to or even exceeded the D0 levels. The chlorophyll fluorescence parameters could not be used to characterize the recoverability of metabolism in tea trees. The electrical characteristics quickly reflected the response of the water metabolism in plants to environmental changes, and the fusion of electrical characteristics and photosynthetic characteristics was able to more quickly, accurately, and comprehensively reflect the response of water metabolism to temporary karst drought.

## 1. Introduction

Guizhou is a typical karst region that is characterized by shallow and discontinuous soil layers, slow soil formation, high limestone porosity, poor water retention capacity, calcium-rich soil, high infiltration rate and extremely fragile ecological environment, etc., with an exposed carbonate rock area of 130,000 km^2^, accounting for more than 70% of the total area of the province. The region receives abundant rainfall, with an average annual precipitation of about 1300 mm.^1–5^ Due to its typical dualistic surface-subsurface hydrological system, rainwater quickly seeps into the underground gap under the action of seepage, resulting in an abundance of groundwater and shortages of surface water. The residual surface moisture can only maintain the soil moisture for a few days, resulting in frequent temporary droughts in the soil; as a result, plants in the region are often subjected to karst drought.^6,7^ Under drought stress, calcium interacts with intracellular Ca^2+^ receptor-calmodulin binding and participates in the sensing, transmission, response, and expression of stress signals, thereby alleviating the effects of environmental stress on plant cell membranes.^8^ As plants have grown in alternatively wet and dry environments over a long period of time, they have evolved different physiological mechanisms and strategies for adapting to changes in their habitats. Different plants respond differently to drought stress, and different cultivars of the same plant species also have very different drought tolerance and recovery abilities in the process of drought and rewatering.^9^ The tolerance of plants to drought and the recoverability of metabolism in plants after rewatering are crucial for adaptation to environments that undergo karst drought.^10^

Water is an abiotic factor that plants depend on for survival, and frequent temporary droughts limit the survival, growth, and distribution of plants in karst areas.^6^ Plants synthesize organic matter and energy through photosynthesis, which is the most important life activity of plants, and chlorophyll is an important pigment in plant photosynthesis. On the one hand, drought stress directly reduces photosynthesis, and on the other hand, it leads to chlorophyll decomposition, which indirectly decreases photosynthesis.^11^ The integrity of photosynthetic organs can be characterized by chlorophyll fluorescence parameters, where Fv/Fm is the maximum photochemical quantum yield of PS II; it remains at around 0.8 under non-stress conditions and significantly decreases under stress conditions.^12^ Most of the water absorbed by plant roots (about 97%~99%) is dissipated through transpiration, and only a small part (about 1%~3%) is stored in plant leaf cells for photosynthesis, growth and other metabolic activities, which shows that this small amount of water is particularly important.^13^ This small amount of water stored in cells can be defined as intracellular water, and the majority of the water dissipated through transpiration is called extracellular water. Many scholars have studied intracellular water mainly through photosynthesis,^14–16^ but photosynthesis makes up only a part of intracellular water; it cannot fully reflect the dynamic changes in intracellular water due to water deficit, and the transport and metabolism of water stored in plant leaf cells are unknown. Electrical parameters enable rapid and effective assessment of intracellular water metabolism.^17,18^

Almost all life activities in plants involve charge separation, electron motion, and proton and dielectric transport.^19^ Plant cells and organelles can be considered as capacitors that can conduct electricity based on electrical principles; organelles with certain structures and functions, e.g., vacuoles and the cytoplasm, occupy most of the intracellular space and can be regarded as resistors. Phospholipids and proteins are the main components of plant cell membranes, and the phospholipid bilayer of the cell membrane is a bilayer with high electrical resistivity because the lipid bilayer of the membrane represents the dielectric phase of the capacitor, which separates the two aqueous conducting phases on either side, resulting in the inability of ions and charged molecules to move freely across the membrane, some of which can only cross the membrane under normal biological conditions by means of specialized protein complexes (pores, channels, pumps, and transporter proteins). Therefore, it is able to store electrical charge.^20–22^ The outer and inner cell membrane are filled with extracellular and intracellular solutions, which can be regarded as conductors. The cell membrane can be regarded as a capacitor; its phospholipid bilayer is equivalent to the bipolar plates of a capacitor, while the ions, ionic groups, and electric dipoles of cells can be regarded as the electrolytes of a capacitor.^23^ Due to the strict selective permeability of the cell membrane of cells, changes in water content inevitably lead to changes in electrolyte concentrations inside and outside the cell, changes in electrical parameters, such as physiological capacitance, resistance, and impedance, due to changes in electrolyte concentration.

Some scholars have used electrophysiology to diagnose the water status of plants. Yu et al. conducted a study on the growth of *Broussonetia papyrifera* and *Morus alba* in waterfront areas and water-deficient areas, and they found that the electrical parameters, capacitance (C), resistance (R), and impedance (Z) of leaves can characterize the response of plants to soil moisture.^13^ Zhao et al. found that a simulated karst drought reduced the intracellular water-holding capacity and water transfer rate of plants and improved the water utilization efficiency of *Lonicera japonica* and *Parthenocissus quinquefolia*.^24^ More and more scholars are applying plant electrical parameters to the study of the water metabolism of plants.^23,25^ However, it is still unclear how the intracellular and extracellular water in tea trees coordinate under water deficit, which remains to be revealed.

The tea tree (*Camellia sinensis* (L.)), which belongs to the genus *Camellia* of the Theaceae family, is a subtropical perennial evergreen plant that is renowned for its numerous benefits, including relieves fatigue, preventing cancer, and lowering blood pressure. It is the second most consumed nonalcoholic beverage in the world.^26,27^ Tea trees are grown in 60 countries and regions around the world.^28^ Tea tree planting in China has a long history of more than 5000 years and rich resources. At present, there are 23 genera and more than 380 species of Theaceae plants, which originated in southwestern China. Theaceae plants are a traditional economic plant in southern China and constitute one of the signature industries and significant economic crops in Guizhou Province.^29–31^

In view of the frequent temporary droughts in karst regions, the tea industry in Guizhou faces challenges. However, research indicates that these droughts are short-lived and can be alleviated by timely rainfall.^32^ It is, thus, crucial to examine the adaptability of tea plants to variations in water availability. This study aims to investigate how various tea varieties regulate photosynthesis and intracellular water metabolism and transportation under water-limited conditions. How do different tea varieties respond to intracellular water transportation and utilization strategies at varying levels of drought? Based on the ample tea tree resources in the karst region of Guizhou, experiments on potted plants under simulated natural drought and subsequent rewatering were conducted in this study. Three different tea varieties were used to investigate changes in the electrical and photosynthetic characteristics of tea plants under temporary drought and rewatering conditions. The physiological response mechanisms of the different tea varieties to the temporary drought and rewatering process were explored, leading to a better understanding of tea trees’ drought tolerance. This study aims to provide valuable insights into the selection of crop varieties and drought-resistant breeding in karst regions.

## 2. Materials and methods

### 2.1. Experimental materials and experimental design

**Figure 1.**
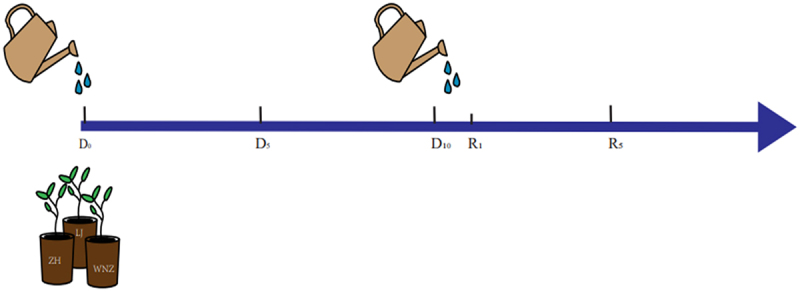
Drought and rehydration treatment diagrams for three tea tree varieties.

The purchased test materials were three annual tea plant seedlings with different drought resistance, namely Zhonghuang No. 2 (ZH), Wuniuzao (WNZ) and Longjing No. 43 (LJ). They were planted in early January 2023. Cultivation containers were polyethylene plastic buckets, which were managed for watering and weeding as per routine. The soil was taken from a karstic landscape soil near the experimental site, with a pH value of 7.80, a total carbon content of 28.57/(mg/g) and a total nitrogen content of 2.27/(mg/g). After a four-month period of slow seedling growth, seedlings with relatively consistent growth potential and healthy and disease-free tea seedlings were selected in late May 2023. The experimental treatments are shown in Figure 1: 1.The control condition was soil moisture content of 40%~45% (D0); 2. Keeping D0 without watering for 5 days (D5); 3. Keep D0 without watering for 10 days (D10); 4. D10 Rehydrate to the first day after D0, (R1); 5. D10 rehydration to the fifth day after D0, (R5).

The experiments were conducted in a greenhouse at the Guizhou Vocational College of Agriculture in Qingzhen City, Guiyang City, Guizhou Province (26°34′N，106°25′E). The photosynthetic photon flux density (PPFD) in the greenhouse was 500 μmol·m^−2^·s^−1^, the daily light time was 12 h, the temperature was 22 ± 2°C, the minimum temperature at night was 16°C, and the indoor relative humidity was 60%~65%.

### 2.2. Determination of soil water content and chlorophyll content

Soil water content was determined at 9:00 to 11:00 a.m. using a HydraGO probe, which was placed close to the roots of the tea seedlings and about 8 cm deep into the soil to determine the soil water content. The chlorophyll content of the first and second leaves of fully developed new shoots was determined using a hand-held SPAD-502Plus chlorophyll meter (Konica Minolta, Tokyo, Japan).

### 2.3. Determination of photosynthesis and chlorophyll fluorescence parameters

The net photosynthetic rate (Pn), stomatal conductance (gsw), transpiration rate (Tr) and intercellular CO_2_ concentration (C_i_) of the first and second fully developed leaves of tea shoots were measured by Li-6800 photosynthesis analyzer (LI-COR Inc., Lincoln, NE, USA) from 9:00 to 11:00 in the morning, and water use efficiency (WUE) was calculated according to the formula WUE=Pn/Tr. The measurement conditions were a flow rate of gas of 500 mmol s^−1^, photosynthetic active radiation of 500 µmol m^−2^ s ^−1^, leaf temperature of 27◦C, CO_2_ concentration of 400 µmol mol^−1^ and humidity of 55%.

The chlorophyll fluorescence parameters of the first and second fully developed leaves of tea shoot were determined by Li-6800 photosynthesis analyzer from 13:00 to 14:00. Before the measurement, tea plants were dark adapted for 1 h, and Fv/Fm was calculated according to the formula Fv/Fm=(Fm-Fo)/Fm.

### 2.4. Determination of electrical parameters

The electrical signal of the first and second fully developed leaves of tea shoot was measured from 14:00 to 16:00 in the afternoon. The leaves were clamped between homemade parallel electrode plates and connected to an LCR-6300 (Gwinstek, Taiwan, China) tester. The measurement frequency was 3k Hz and the measurement voltage was 1.5 V. In parallel mode, Z, C and R of tea tree were measured under different clamping forces (1.17N, 2.17N, 3.17N, 5.17N, and 7.17N). 11 ~ 13 data points were collected for each clamping force. Subsequently, foliar Xc and XL were calculated based on Equations (1) and (2), respectively.

(1) $$\mathrm{Xc}=\frac{1}{2\pi \mathrm{fC}}$$

(2) $$\frac{1}{-\mathrm{XL}}=\frac{1}{Z}-\frac{1}{R}-\frac{1}{\mathrm{Xc}}$$

Note: Xc: capacitive reactance, π: 3.1416,f:frequency, C: capacitance, XL: inductive resistance, Z: impedance, R: resistance.

### 2.5. Calculation of electrical parameters

The physiological capacitance (C), physiological resistance (R), physiological impedance (Z), physiological capacitance (XC), and physiological susceptibility (XL) of plant leaves can be calculated by referring to the previous research method of Zhang et al.^23^

The fitting equations for the blade clamping force to R, Z, Xc, XL and C are equations (3)–(7):

(3) $$R=\;y_{1}+k_{1}e^{-\;b_{1}F}$$

(4) $$Z=\;y_{2}+k_{2}e^{-\;b_{2}F}$$

(5) $$\mathrm{Xc}=\;y_{3}+k_{3}e^{-\;b_{3}F}$$

(6) $$\mathrm{XL}=\;y_{4}+k_{4}e^{-\;b_{4}F}$$

(7) $$C=y_{{\;}_{0}}\;+\;\mathrm{hF}$$

When the clamping force (F = 0N) is applied, the intrinsic resistance (IR), intrinsic impedance (IZ), intrinsic capacitive reactance (IXc), intrinsic inductive reactance (IXL), intrinsic capacitance (ICP), and specific effective thickness (d) can be calculated accordingly from Equations (3)-(7) to obtain Equations (8)-(13):

(8) $$\mathrm{IR}\;=y_{1}\;+k_{1}$$

(9) $$\mathrm{IZ}=y_{2}+k_{2}$$

(10) $$\mathrm{IXc}\;=y_{3}+k_{3}$$

(11) $$\mathrm{IXL}\;=y_{4}+k_{4}$$

(12) $$\mathrm{ICP}\;=\frac{1}{2\pi \;\mathrm{fIXc}}$$

### 2.6. Definition of intracellular water parameters

The intracellular water metabolism parameters of tea leaves were calculated according to equation (13)-(16), including intracellular water-holding capacity (IWHC), intracellular water-use efficiency (IWUE), intracellular water retention time (IWHT) and water transfer rate (IWTR):

(13) $$\mathrm{IWHC}\;=\sqrt{{\left(\mathrm{ICP}\right)}^{3}}$$

(14) $$\mathrm{IWUE}\;=\frac{d}{\mathrm{IWHC}}$$

(15) IWHT=ICP× IZ

(16) $$\mathrm{IWTR}\;=\frac{\mathrm{IWHC}}{\mathrm{IWHT}}$$

### 2.7. Definition of nutritional parameters

The relative nutrient flux per unit area (UNF), active transport flow of nutrient (UAF), nutrient transfer rate (NTR), nutrient transport capacity (NTC) and nutrient active transport capacity (NPC) were calculated according to equation (17)–(21):

(17) $$\mathrm{UNF}\;=\frac{\mathrm{IR}}{\mathrm{IXc}}+\frac{\mathrm{IR}}{\mathrm{IXL}}$$

(18) NTR=WTR

(19) NTC=UNF× NTR

(20) $$\mathrm{UAF}\;=\frac{\mathrm{IXc}}{\mathrm{IXL}}$$

(21) NPC=UAF× NTR

### 2.8. Data analyses

Microsoft Excel 2010 software was used for data statistics; Software IBM SPSS 21.0 was used for one-way ANOVA and Duncan’s method for multiple comparisons (*p* < 0.05); All the data were expressed as mean ± standard deviation (Mean ± SD) (*n* = 3); and plotted using Origin2018 software.

## 3. Results

### 3.1. Changes in soil water content

**Figure 2.**
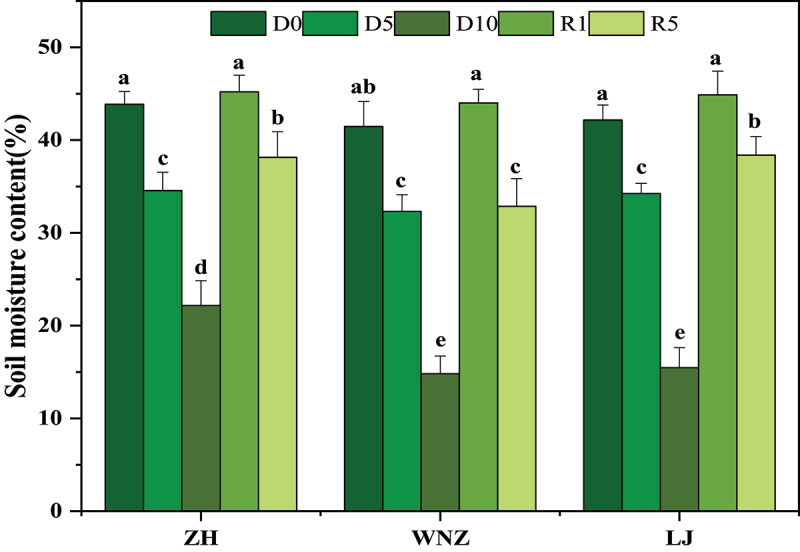
Soil water content of three tea tree varieties under five phase treatments. Data are expressed as Mean ± SD, *n* = 3. The difference of different lowercase letters above the error bar was significant at 5% level (*p* < 0.05).

As shown in Figure 2, the soil water content of the three tea tree varieties in the D0 stage was between 40% and 45%. The soil water content of the three tea cultivars at D5 decreased by 22.11%~18.83% compared with the control group. The soil water content of ZH, LJ and WNZ was 22.17%, 15.47% and 14.8%, respectively. The soil was hardened at D10, and it was difficult for the probe to penetrate 8 cm into the soil for determination; therefore, a rewatering treatment was carried out. After rehydration for 1d (R1), the soil water content was in the range of 40%~45%, which was consistent with the control group. The soil water content decreased again after rehydration for 5 days (R5). The above results showed that the soil water content had a decreasing trend with the extension of the drought treatment time.

### 3.2. Effects of drought and rehydration on the electrical parameters of tea tree leaves

As shown in Table 1, compared with D0, d was significantly reduced in the three tea tree varieties at D5, and no significant changes were observed in ZH and LJ after D10, while WNZ first decreased and then recovered to D0. Drought increased the IR, IZ, IX_C_, and IXL values of the three varieties. The IZ and IX_C_ values of WNZ did not significantly change in the five-phase treatments, and the other two varieties did not reach D0 after rehydration, but the IZ and IX_C_ values of Longjing 43 changed the most in the five-phase treatments. The change in IXL was consistent with that of IR. The ICP value gradually decreased with the severity of the drought, the ICP value of ZH was significantly decreased at D5, and then there were no significant changes. There were no significant changes in ICP of WNZ in the five-phase treatments. The ICP value of LJ, which did not reach the level at D0 after rewatering, was significantly reduced at both D5 and D10. The above results showed that the drought treatment decreased the ICP and increased the IR, IZ, IX_C_, and IXL values of the tea trees, among which Longjing 43 had the largest change, that is, Longjing 43 was the most sensitive to drought and rehydration, followed by Zhonghuang 2, and Wuniuzao was the least sensitive.

**Table 1.** Electrical parameters of ZH, WNZ, and LJ under five-phase treatments.

Electrical parameters	Treatment	ZH	WNZ	LJ
d	D0	25.90 ± 4.32^a^	23.46 ± 1.24^h^	45.75 ± 12.76°
	D5	13.35 ± 2.94^b^	17.04 ± 4.07^i^	23.00 ± 3.01^p^
	D10	12.90 ± 1.80^b^	16.62 ± 1.32^i^	17.54 ± 3.15^p^
	R1	16.14 ± 3.98^b^	13.73 ± 0.52^i^	15.88 ± 1.84^p^
	R5	18.15 ± 5.94^b^	24.08 ± 3.07^h^	24.23 ± 1.37^p^
IR	D0	4.22 ± 0.38^b^	6.20 ± 3.21^i^	2.83 ± 0.79^p^
	D5	16.30 ± 7.64^ab^	13.56 ± 6.40^hi^	6.10 ± 3.04^p^
	D10	14.65 ± 8.71^ab^	19.22 ± 6.76^h^	17.52 ± 2.95°
	R1	9.34 ± 2.38^ab^	12.92 ± 6.06^hi^	7.76 ± 3.50^p^
	R5	21.05 ± 10.98^a^	19.29 ± 4.69^h^	5.34 ± 1.20^p^
IZ	D0	1.10 ± 0.13^b^	1.33 ± 0.27^h^	0.62 ± 0.06^r^
	D5	1.63 ± 0.12^ab^	1.52 ± 0.23^h^	1.03 ± 0.13^q^
	D10	2.14 ± 0.69^a^	1.68 ± 0.17^h^	1.85 ± 0.14°
	R1	1.65 ± 0.31^ab^	1.59 ± 0.11^h^	1.32 ± 0.21^p^
	R5	1.86 ± 0.23^a^	1.74 ± 0.36^h^	1.37 ± 0.13^p^
IX_C_	D0	1.14 ± 0.14^b^	1.37 ± 0.26^h^	0.64 ± 0.06^r^
	D5	1.64 ± 0.12^ab^	1.53 ± 0.23^h^	1.05 ± 0.13^q^
	D10	2.18 ± 0.70^a^	1.68 ± 0.18^h^	1.86 ± 0.15°
	R1	1.67 ± 0.32^ab^	1.61 ± 0.10^h^	1.34 ± 0.20^p^
	R5	1.87 ± 0.22^a^	1.74 ± 0.36^h^	1.42 ± 0.13^p^
ICP	D0	47.11 ± 5.95^a^	39.72 ± 7.42^h^	83.46 ± 7.33°
	D5	32.46 ± 2.32^b^	35.18 ± 4.85^h^	51.02 ± 6.62^p^
	D10	26.19 ± 8.55^b^	31.77 ± 3.62^h^	28.67 ± 2.21^r^
	R1	32.41 ± 5.63^b^	33.10 ± 1.96^h^	40.26 ± 5.55^q^
	R5	28.67 ± 3.49^b^	31.24 ± 5.88^h^	37.52 ± 3.47^qr^
IXL	D0	4.86 ± 0.42^b^	6.59 ± 3.32^i^	3.19 ± 0.85^q^
	D5	17.44 ± 7.92^ab^	14.35 ± 6.48^hi^	6.67 ± 3.09^pq^
	D10	15.47 ± 8.43^ab^	20.02 ± 6.37^h^	18.65 ± 3.30°
	R1	10.23 ± 2.44^ab^	13.69 ± 5.97^hi^	8.44 ± 3.52^p^
	R5	21.50 ± 10.27^a^	20.07 ± 4.47^h^	6.13 ± 1.22^pq^

Data are expressed as Mean ± SD, *n* = 3. The different lowercase letters have significant differences at the 5% level (*p* < .05 according to Duncan’s test), and abc et al. represent the differences between ZH values; hij et al. represent the difference between WNZ values; opq et al. represent the difference between LJ values; d:specific effective thickness, IR:intrinsic resistance, IZ:intrinsic impedance, IX_C_:intrinsic capacitive reactance, ICP:intrinsic capacitance, IXL:intrinsic inductive reactance.

### 3.3. Effects of drought and rehydration on the intracellular water parameters of tea tree leaves

According to the findings in Table 2, compared with those at D0, the IWHC and IWTR values of ZH and LJ were significantly decreased at D5, and there were no significant changes in ZH at D10, while LJ still significantly decreased, and neither reached the control level after rehydration. The IWHC and IWTR values of WNZ did not significantly change in the five-phase treatments. Drought increased the IWHT values of the three tea cultivars, indicating that the plants responded to drought by increasing their IWHT and decreasing their IWTR under drought stress; there were no significant changes in ZH and WNZ after rewatering, while LJ showed a minimum at R5. Meanwhile, drought increased the IWUE value, ZH did not significantly change in the five-phase treatments, and the WNZ value changed in the same way as the value of ZH, but the maximum value of WNZ appeared at R5. The IWUE value of LJ was significantly larger than that at D0 and D5 at D10, and it was significantly reduced after rehydration and then significantly increased. The above results showed that the D5 and D10 treatments reduced the IWHC and IWTR of tea trees and curbed the intracellular water metabolism of their leaves; only WNZ reached the control level after rehydration, indicating that it had the strongest recovery due to rehydration, i.e., WNZ was the least sensitive to water deficit, followed by ZH, and LJ was the most sensitive in relative terms.

**Table 2.** Intracellular water use parameters of ZH, WNZ, and LJ under five-phase treatments.

Electrical parameters	Treatment	ZH	WNZ	LJ
IWHC	D0	324.00 ± 62.10^a^	252.51 ± 70.40^h^	763.88 ± 100.57°
	D5	185.19 ± 19.88^b^	209.67 ± 42.32^h^	365.92 ± 71.65^p^
	D10	127.61 ± 66.42^b^	179.68 ± 31.13^h^	153.71 ± 17.77^q^
	R1	185.89 ± 46.99^b^	190.58 ± 16.79^h^	256.69 ± 51.76^pq^
	R5	154.08 ± 28.20^b^	176.21 ± 48.10^h^	230.31 ± 32.01^q^
IWTR	D0	6.33 ± 1.16^a^	4.91 ± 1.43^h^	14.81 ± 2.08°
	D5	3.52 ± 0.38^b^	3.99 ± 0.82^h^	7.03 ± 1.39^p^
	D10	2.64 ± 1.29^b^	3.40 ± 0.57^h^	2.92 ± 0.33^q^
	R1	3.56 ± 0.90^b^	3.64 ± 0.34^h^	4.93 ± 1.03^pq^
	R5	2.92 ± 0.54^b^	3.33 ± 0.90^h^	4.50 ± 0.64^q^
IWHT	D0	51.21 ± 0.49^b^	51.51 ± 0.79^i^	51.60 ± 0.52^p^
	D5	52.61 ± 0.17^a^	52.59 ± 0.35^h^	52.04 ± 0.55o^p^
	D10	52.27 ± 0.64^a^	52.82 ± 0.22^h^	52.72 ± 0.14°
	R1	52.14 ± 0.45^a^	52.37 ± 0.84^hi^	52.13 ± 0.56o^p^
	R5	52.87 ± 0.51^a^	52.86 ± 0.19^h^	51.17 ± 0.76^p^
IWUE	D0	0.08 ± 0.02^a^	0.10 ± 0.02^i^	0.06 ± 0.03^p^
	D5	0.07 ± 0.01^a^	0.08 ± 0.01^i^	0.06 ± 0.02^p^
	D10	0.11 ± 0.04^a^	0.09 ± 0.02^i^	0.11 ± 0.01°
	R1	0.09 ± 0.01^a^	0.07 ± 0.00^i^	0.06 ± 0.01^p^
	R5	0.12 ± 0.02^a^	0.14 ± 0.04^h^	0.11 ± 0.02°

Data are expressed as Mean ± SD, *n* = 3. The different lowercase letters have significant differences at the 5% level (*p* < .05 according to Duncan’s test). IWHC: intracellular water-holding capacity, IWTR:water transfer rate, IWHT:intracellular water-holding time, IWUE:intracellular water use efficiency.

### 3.4. Effects of drought and rehydration on the nutrient parameters of the three tea cultivars

As shown in Table 3, drought increased the UNF values of the three tea cultivars, and the UNF value of ZH did not return to D0 after rewatering; the UNF value of WNZ did not significantly change among the five-phase treatments. That of LJ was significantly larger than in the remaining four treatments at D10, and there were no significant difference among the remaining four treatments, suggesting that the UNF value of WNZ was the least sensitive to the drought rewatering treatments, followed by LJ and ZH. The NTC values of ZH and WNZ were not significantly different in the five-phase treatments; drought significantly reduced the NTC value of LJ, which was significantly smaller than that at D0 after rewatering, indicating that the NTC values of ZH and WNZ were not significantly affected by water. Drought reduced the UAF values of all three varieties, with all recovering slightly at R1, and only LJ recovering to D0 at R5; the NPC values of all three varieties were significantly reduced at D5, and none of them significantly changed thereafter.

**Table 3.** Nutrient parameters of ZH, WNZ, and LJ under five-phase treatments.

Electrical parameters	Treatment	ZH	WNZ	LJ
UNF	D0	4.61 ± 0.53^b^	5.25 ± 1.49^h^	5.26 ± 0.97^p^
	D5	10.72 ± 3.98^ab^	9.59 ± 3.05^h^	6.69 ± 2.76^p^
	D10	7.70 ± 3.99^ab^	12.78 ± 5.66^h^	10.37 ± 1.43°
	R1	6.53 ± 1.41^ab^	8.90 ± 3.55^h^	6.56 ± 1.72^p^
	R5	12.08 ± 4.96^a^	12.48 ± 4.41^h^	4.61 ± 0.70^p^
NTC	D0	29.52 ± 8.57^a^	24.62 ± 3.33^h^	76.90 ± 9.26°
	D5	36.94 ± 10.24^a^	37.09 ± 9.30^h^	46.44 ± 17.86^p^
	D10	19.60 ± 10.59^a^	45.60 ± 28.43^h^	30.27 ± 5.84^pq^
	R1	23.49 ± 9.18^a^	31.91 ± 11.59^h^	31.21 ± 3.05^pq^
	R5	34.59 ± 12.73^a^	43.64 ± 24.75^h^	20.70 ± 3.90^q^
UAF	D0	0.23 ± 0.03^a^	0.21 ± 0.05^h^	0.21 ± 0.04°
	D5	0.10 ± 0.03^b^	0.12 ± 0.04^i^	0.18 ± 0.06^op^
	D10	0.16 ± 0.07^ab^	0.09 ± 0.03^i^	0.10 ± 0.02^p^
	R1	0.17 ± 0.04^ab^	0.14 ± 0.07^hi^	0.17 ± 0.04^op^
	R5	0.10 ± 0.05 ^b^	0.09 ± 0.03^i^	0.24 ± 0.04°
NPC	D0	1.47 ± 0.17^a^	1.10 ± 0.52^h^	3.15 ± 1.16°
	D5	0.37 ± 0.15^b^	0.49 ± 0.24^i^	1.25 ± 0.55^p^
	D10	0.44 ± 0.33^b^	0.30 ± 0.07^i^	0.30 ± 0.06^p^
	R1	0.59 ± 0.19^b^	0.51 ± 0.29^i^	0.86 ± 0.37^p^
	R5	0.29 ± 0.14^b^	0.29 ± 0.06^i^	1.06 ± 0.24^p^

Data are expressed as Mean ± SD, *n* = 3. The different lowercase letters have significant differences at the 5% level (*p* < .05 according to Duncan’s test). UNF: nutrient flux per unit area, NTC:nutrient transport capacity, UAF:active transport flow of nutrient, NPC:nutrient active transport capacity.

### 3.5. Effects of drought and rehydration on the photosynthetic characteristics of the three tea cultivars

**Figure 3.**
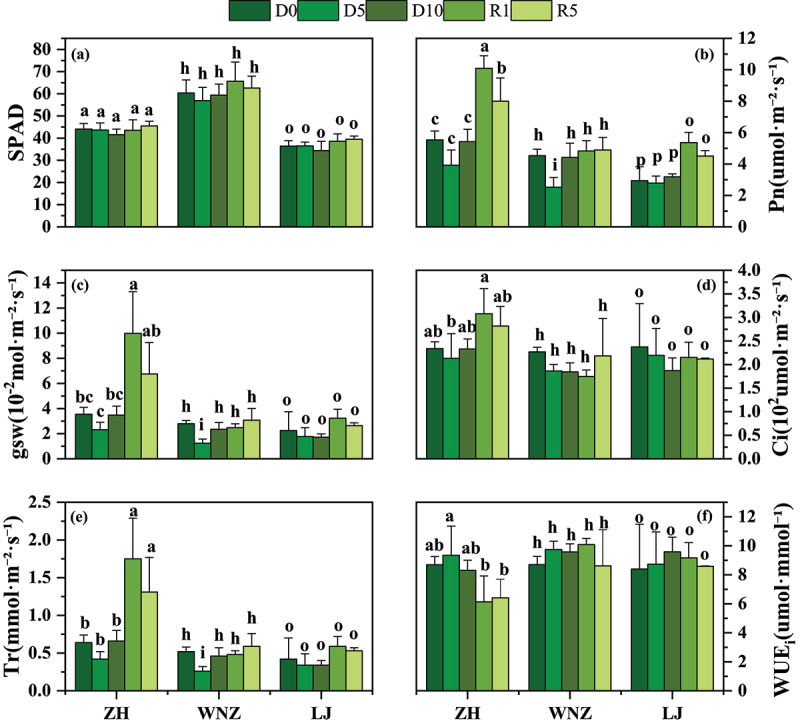
Photosynthetic characteristics of ZH, WNZ and LJ under five phase treatments. Data are expressed as Mean ± SD, *n* = 3. The different lowercase letters have significant differences at the 5% level (*p* < .05 according to Duncan’s test). SPAD:relative chlorophyll content, Pn:net photosynthetic rate, gsw:stomatal conductance, Ci: intercellular carbon dioxide concentration, Tr:rate of transpiration, WUE_i_: instantaneous water-use efficiency.

As can be seen in Figure 3a, the SPAD values of the three tea tree varieties did not significantly change in the five-phase treatments. The Pn, gs, and Tr values of ZH had no significant changes before D10. Compared with D0, the Pn, gs, and Tr values at R1 were significantly increased by 82.13%, 150%, and 173.44%, respectively, while those at R5 were increased by 44.40%, 75%, and 104.69%. Compared with D0, the Pn, gs, and Tr values of WNZ significantly decreased at D5, and then the values had no significant changes with respect to D0. Before D10, the Pn value of LJ did not significantly change. At R1 and R5, the Pn value of LJ was significantly increased by 82.31% and 53.40%, respectively, compared with that in the D0 treatment, and the gs and Tr values did not significantly change in the five-phase treatments. As shown in Figure 3d, from D0 to D10, the intercellular CO_2_ concentration (C_i_) in ZH first decreased and then slightly increased; no significant changes were observed before D10, and no significant changes were observed after rehydration. The C_i_ values of WNZ and LJ gradually decreased with the intensification of drought, but the change was not obvious in the five-phase treatments. As shown in Figure 3f, the WUE_i_ values of ZH slightly increased from D0 to D5 and then gradually decreased; drought slightly increased the WUE_i_ values of WNZ and LJ but did not change them significantly in the five-phase treatments. Overall, only WNZ was damaged during the 10 days of drought, and ZH had the strongest photosynthetic recovery ability after water resumption, followed by LJ and WNZ.

### 3.6. Effects of drought and rehydration on chlorophyll fluorescence parameters of the three tea cultivars

**Figure 4.**
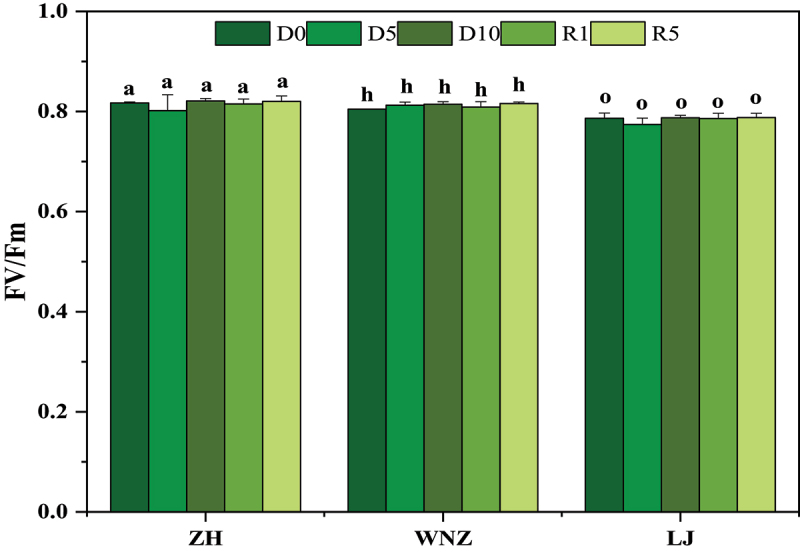
Chlorophyll fluorescence parameters of ZH, WNZ and LJ under five phase treatments. Data are expressed as Mean ± SD, *n*=3. The different lowercase letters have significant differences at the 5% level (*p*<0.05 according to Duncan’s test). Fv/Fm: maximum photochemical efficiency.

As Figure 4 shows, there were no the significant differences in Fv/Fm among the three tea tree varieties in the five-phase treatments, indicating that the degree of water deficit in this experiment did not touch the critical point of the change in Fv/Fm.

### 3.7. Correlation between photosynthetic characteristics and electrical parameters in tea trees

As shown in Table 4, there was no correlation between gas exchange parameter and electrical parameters, intracellular water, and nutrient parameters in the three tea tree varieties, except for NTC in LJ, indicating that there is no direct correlation between intracellular water metabolism and extracellular water metabolism in this study.

**Table 4.** Pearson’s correlation coefficients between gas exchange parameter and electrical parameters of ZH, WNZ, and LJ.

Parameters	ZH			WNZ			LJ		
	Tr	Pn	gsw	Tr	Pn	gsw	Tr	Pn	gsw
d	–0.180	–0.065	–0.177	0.512	0.230	0.502	–0.338	–0.499	–0.317
IR	0.017	0.008	–0.034	–0.072	0.042	–0.083	–0.326	–0.135	–0.333
IZ	0.195	0.122	0.175	0.070	–0.077	0.055	–0.076	0.205	-0.114
IXC	0.196	0.120	0.178	0.078	–0.074	0.063	–0.061	0.221	–0.102
ICP	–0.275	–0.191	–0.253	–0.069	0.037	–0.047	0.009	–0.330	0.056
IXL	0.010	–0.003	–0.039	–0.072	0.034	–0.084	–0.321	–0.126	–0.329
IWHC	–0.281	–0.197	–0.259	–0.067	0.026	–0.042	0.013	–0.336	0.060
IWHT	0.065	0.075	0.022	–0.078	–0.027	–0.095	–0.443	–0.244	–0.405
IWTR	–0.278	–0.196	–0.255	–0.060	0.024	–0.035	0.024	–0.329	0.071
IWUE	0.151	0.165	0.124	0.470	0.129	0.452	–0.293	–0.106	–0.354
UNF	–0.071	–0.056	–0.120	–0.085	0.098	–0.096	–0.484	–0.319	–0.465
NTC	–0.337	–0.251	–0.363	–0.100	0.146	–0.105	–0.313	–0.545*	–0.248
UAF	0.018	0.008	0.059	0.110	0.030	0.123	0.498	0.278	0.467
NPC	–0.173	–0.123	–0.144	0.043	–0.003	0.068	0.238	–0.172	0.269

*reprsents that correlation is significant at the 0.05 level (two-tailed). d:specific effective thickness, IR:intrinsic resistance, IZ:intrinsic impedance, IX_C_:intrinsic capacitive reactance, ICP:intrinsic capacitance, IXL:intrinsic inductive reactance, IWHC:intracellular water-holding capacity, IWTR:water transfer rate, IWHT: intracellular water-holding time, IWUE:intracellular water use efficiency, UNF: nutrient flux per unit area, NTC:nutrient transport capacity, UAF:active transport flow of nutrient, NPC:nutrient active transport capacity, Pn:net photosynthetic rate, gsw: stomatal conductance, Tr:rate of transpiration.

## 4. Discussion

### 4.1. Electrical parameters and recoverability due to drought rehydration of tea trees

In a state of optimal health, plants’ physiological activities are more vigorous, and their cells are able to store more ions, ionic groups, and electric dipoles, which can be defined as a generalized charging phenomenon.^33,34^ Therefore, the healthier the plant and the less leakage through plant leaves, the higher the ICP value and the lower the IR, IZ, IX_C_, and IXL values. In this study, the decrease in soil water content resulted in a decrease in the ICP of tea leaves and an increase in their IR, IZ, IXC, and IXL values, indicating that water deficit can inhibit healthy growth, which is consistent with the research results of Yu et al.^13^ After rehydration, the d, IZ, IX_C_, and ICP values of WNZ were able to recover to the D0 level, and the IR value of LJ also slightly recovered. Although the electrical parameters of ZH did not return to the levels at D0, the amplitude of changes in the electrical parameters of ZH was less than that of LJ in the five-phase treatments, and the amplitude of the changes in WNZ was the smallest, suggests that WNZ is less sensitive to drought stress. The above findings indicate that WNZ is more drought tolerant than the other two varieties. Water metabolism and nutrient transfer within plant cells can reflect plant growth and development. Zhang et al. found in a study of *Broussonetia papyrifera* growing in different soils (agricultural soil and moderately rocky desertification soil) that plants in good habitats had higher ICP, IWHC, WTR, NTR, and NTC values, while they had lower values of IR, IZ, IX_C_, and IXL.^23,35^ In this study, the D5 treatment significantly decreased the IWHC and IWTR of the leaves of the three tea tree varieties; that of LJ continued to significantly decrease at D10, and the other two varieties had no significant changes; thus, drought inhibited the water metabolism in the tea trees. After rehydration, only WNZ recovered to the D0 level, which showed that the recovery of the water metabolism of WNZ was stronger than that of the other two varieties, indicated that WNZ was more drought tolerant than the other two tea tree varieties. When the soil water content was high, the roots absorbed too much water, and the intracellular water provided sufficient water, so the water transfer rate was high, and nutrients were transferred with the transfer of water. Therefore, IWHC, IWTR, and NTR maintained a high level when the water was sufficient. In a water deficit, less water is absorbed by the root system, resulting in a lower IWHC value; a plant maintains growth by extending its IWHT and reducing its WTR or NTR. The water transport of plants is diverse; the intracellular water metabolism of different plants has different responses to the environment, and the same plant in different growing environments has different responses to those environments. Zhang et al. found four different water transport strategies in their studies on potato, pepper, and *B. papyrifera* in different growing environments^23^: (1) high IWHC, high IWTR, and high IWUE for potato; (2) medium IWHC, medium IWTR, and low IWUE for *B. papyrifera* growing in agricultural soils; (3) low IWHC, low IWTR, and low IWUE for *B. papyrifera* growing in moderately rocky desertification soil; (4) medium IWHC, medium IWTR, and high IWUE for chili. With reference to their findings, only one water transport strategy was found in this study, namely, that of medium IWHC, medium IWTR, and low IWUE, which was consistent with the water transport strategy of *B. papyrifera* grown in agricultural soils.

Plant nutrient transport status can also reflect plant growth. In this study, it was observed that drought elevated the UNF value and decreased the NTC, UAF, and NPC values in tea plants, but the nutrient parameters of some varieties could be restored to the D0 levels after rehydration. In general, the recoverability of the nutrient parameters of WNZ was greater than that of the other two varieties. Plants not only have different water transport strategies, but also have different nutrient transport strategies. Four nutrient transport strategies were identified in a previous study by Zhang et al.: (1) low UNF, high NTR, and high NTC (e.g., *Ipomoea batatas* L, *Solanum tuberosum* L., and *B. papyrifera* grown in agricultural soil); (2) high UNF, low NTR, and low NTC (e.g., *B. papyrifera* grown in moderately rocky desertification soils, *Senecio scandens* Buch.-Ham. ex D., and *Capsicum annuum* L.); (3) high UNF, low NTR, and high NTC (e.g., *Rhus chinensis* Mill.); (4) low UNF, high NTR, and low NTC (e.g., *Toona sinensis*).^35^ Referring to their findings, one nutrient transport strategy, i.e., that of high UNF, low NTR, and high NTC, was found in this study, and it was consistent with that of *R. chinensis* Mill. In conclusion, as far as the present study is concerned, only one water transport strategy and one nutrient transport strategy were found in tea trees, and both the water metabolism and nutrient transport parameters characterized the restorative nature of the plants.

### 4.2. Relationship between intracellular water metabolism and the photosynthetic characteristics of tea trees

There were no significant changes in the SPAD content in the five-phase treatments. The chlorophyll fluorescence parameters can reflect the absorption, transfer, and conversion of light energy in leaves, and they are an internal probe for studying the relationship between photosynthesis and the environment; the degree of change in Fv/Fm can be used to identify the ability of plants to tolerate drought.^36^ No significant differences were observed in this study, indicating that the degree of water deficit in this experiment did not reach the critical point of change in Fv/Fm values, suggesting that the Fv/Fm values were not sensitive to these treatments.

Electrophysiology is closely related to photosynthesis, Sukhova et al. showed that electrical signals induced photosynthesis response and increased ATP content in plant leaves, and when long-term inactivation of photosynthesis occurs electrical signals induced stomatal closure of leaves; whereas, Table 4 showed that there was no correlation between electrical parameters and photosynthetic characteristics in the three tea tree cultivars, which might be due to the fact that the response of electrical parameters was greater than that of photosynthetic characteristics under water deficit, whereas the response of electrical parameters was less than that of photosynthetic characteristics after rehydration. Inconsistency in the response phases of electrical and photosynthetic characteristics resulted in no correlation between electrical and photosynthetic characteristics.^37,38^

Proper drought can stimulate stomatal opening and improve drought resistance, while excessive water hinders root respiration and causes hypoxia in plant tissues, which is not conducive to leaf photosynthesis.^39^ The water metabolism in the leaves of different varieties of plants is different at different levels of water deficit.

**Figure 5.**
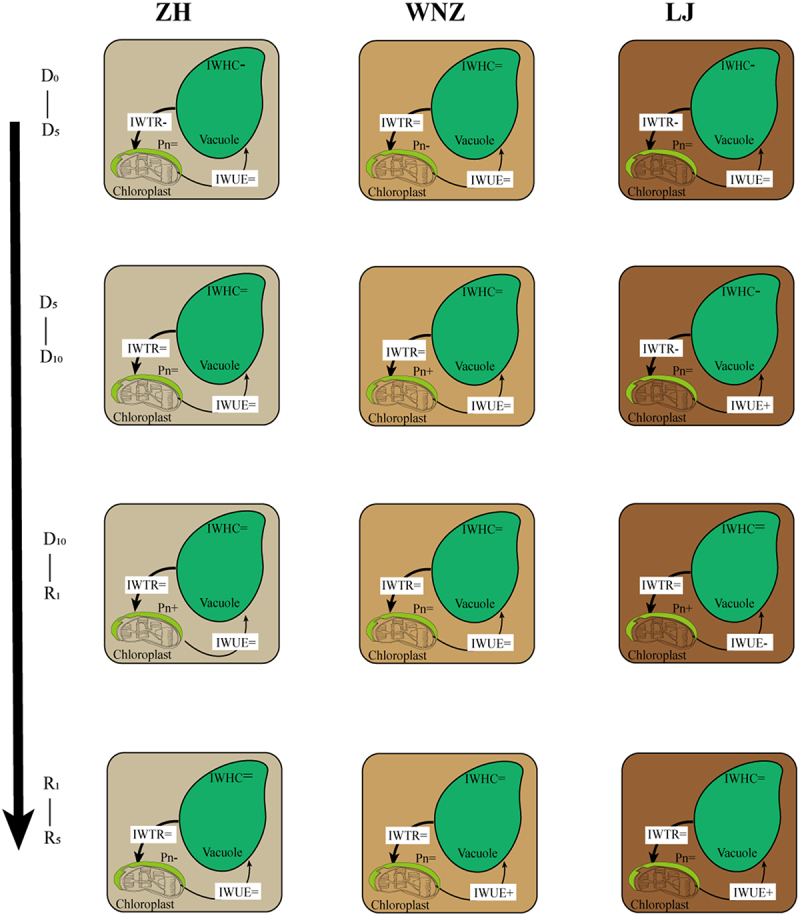
Water metabolism characteristics of three tea tree varieties. IWTR: intracellular water transport rate, IWHC: intracellular water holding capacity, IWUE: intracellular water use efficiency, Pn: net photosynthetic rate. “=”: unchange, “+”: increase, “-”: decrease.

As shown in Figure 5 in the phase of D0-D5, soil water content decreased, for ZH and LJ, intracellular water was first affected, resulting in a decrease in IWHC and IWTR, and the response of extracellular water was slow, so it showed unchanged Pn. WNZ in the phase of D0-D5 were in the state of adequate water, and extracellular water was first affected, whice hinder root respiration and inhibit photosynthesis, while intracellular water was slow to respond, showing unchanged IWHC and IWTR.

In the phase of D5-D10, soil water content continued to decrease, the drought tolerance of ZH trained by drought stress in the previous phase improved, resulting in no change in intracellular water, while the extracellular water metabolism is balanced, and photosynthesis also did not change, but LJ was suppressed by drought, resulting in a continuous decrease in extracellular water, due to the slow response of extracellular water, Pn was unchanged. WNZ in the phase of D5-D10 were in the state of proper water, Pn increases and the intracellular water response is slow, IWHC and IWTR are unchanged. This shows that WNZ is more drought tolerant than the other two varieties, while LJ is least drought tolerant.

In the phase of D10-R1, soil water content increases, for ZH and LJ, extracellular water responds first, the increase in water provides raw material for photosynthesis to take place, Pn increases, the slow response of intracellular water results in IWHC and IWTR remaining unchanged. WNZ was drought tolerant and, conversely, it is not sensitive to water after rehydration, IWHCI, IWTR and Pn were maintained at same levels in the phase of D5-D10, resulting in no change in IWHCI, IWTR and Pn.

In the phase of R1-R5, soil water content decreases, for ZH and LJ, intracellular water was first affected, photosynthesis of ZH was inhibited, resulting in a decrease in Pn, photosynthesis of LJ was unaffected, and IWHC and IWTR were unchanged due to slow intracellular water responses. IWHC, IWTR, and Pn remained unchanged due to the insensitivity of WNZ to water deficit. LJ is more restorative than LJ after rehydration. In summary, it is clear that the tea tree have different intra- and extracellular water metabolism mechanisms during drought and rehydration.

## 5. Conclusion

Drought reduced the ICP value; elevated the IR, IZ, IXc, and IXL values; and decreased the intracellular water metabolism and nutrient transport in tea trees. The net photosynthetic rate could reflect the changes in tea tree under five-phase treatments, while chlorophyll fluorescence parameters did not respond under five-phase treatments. Importantly, the combination of electrical parameters and photosynthetic characteristics provides a more accurate and comprehensive picture of the response of tea trees to changes in arid environments.

## Abbreviations

C: capacitance
Z: impedance
R: resisttance
XC: capacitive reactance
XL: inductive reactance
F: force
d: specific effective thickness
IR: intrinsic resistance
IZ: intrinsic impedance
IXC: intrinsic capacitive reactance
ICP: intrinsic capacitance
IXL: intrinsic inductive reactance
IWHC: intracellular water-holding capacity
IWHT: intracellular water-holding time
IWTR: water transfer rate
IWUE: intracellular water use efficiency
UNF: nutrient flux per unit area
UAF: active transport flow of nutrient
NTC: nutrient transport capacity
NPC: nutrient active transport capacity
